# Fallacies of preoperative lymphoscintigraphy in detecting sentinel node in breast cancer

**DOI:** 10.1186/1477-7819-3-31

**Published:** 2005-05-31

**Authors:** Manoj Pandey, Surya VS Deo, R Maharajan

**Affiliations:** 1 Surgical Oncology, Jawaharlal Nehru Cancer Hospital and Research Centre, Bhopal, India; 2 Surgical Oncology, Institute Rotary Cancer Hospital, All India Institute of Medical Sciences, New Delhi 110 029, India; 3 Department of Nuclear Medicine, West fort Hi-tech Hospital Ltd, Punkunnam, Thrissure 680 002, India

## Abstract

**Background:**

Preoperative lymphoscintigraphy is one of the three methods of evaluating sentinel nodes in patients with breast cancer; however, it has been reported to have a high false negative rate.

**Case presentations:**

We report here two cases where the preoperative lymphoscintigraphy was found to be fallacious. A 44-year-old female with T2N0 breast cancer underwent preoperative lymphoscintigraphy with Tc^99 ^sulfur colloid which failed to show any uptake in axilla or internal mammary chain. Intraoperative scintigraphy with blue dye and hand held gamma probe identified sentinel lymph node in axilla. Another patient with T2N0 lesion underwent preoperative lymphoscintigraphy which showed a sentinel lymph node in axilla and another in supraclevicular fossa. Intraoperative scintigraphy failed to show supraclevicular node however axillary node was correctly identified.

**Conclusion:**

These two cases further strengthen the need to carry out triple test in identification of sentinel lymph node in patients with breast cancer. It also demonstrates the fallacies of preoperative lymphoscintigraphy.

## Background

Metastasis to the axillary lymph node is the single most important prognostic factor in breast cancer. The therapeutic decisions are based on the axillary status. However, in recent past sentinel lymph node identification and biopsy (SLNB) is fast emerging as an alternate to axillary dissection as it avoids the complications of axillary dissection like lymphedema, pain, numbness and limitations of shoulder movements [1,2]. SLNB has been found to be highly predictive of axillary lymph node status with false negative results of less than 5% [3-5]. A number of validity studies have been published however, the question of its oncological safety still awaits the results of randomized clinical trials [6,7].

The sentinel lymph node identification is usually carried out by preoperative localization using Tc^99 ^colloid and gamma camera or by intraoperative localization using hand held gamma probe or by dye technique. Majority of the centers use a combination of techniques and it has been reported that the triple method using all of the above gives the best results [8-10].

Non-visualization of sentinel node at preoperative scintigraphy is a continued problem. Between 3 to 30% of the nodes are reported to be non visualized most of which are subsequently picked up on intraoperative scintigraphy [11-13]. Several factors like age, size of the breast, presence of metastasis, has been proposed to influence the non-visualization [11-17]. We report here two unusual cases of non-visualization or abnormal visualization during preoperative lymphoscintigraphy; both of these cases were subsequently identified and biopsied intraoperatively using a combination of blue dye and hand held gamma probe.

## Case presentation

### Case 1

A 44-year-old female presented with 2 months history of a progressive lump in right breast. She gave a past history of noncyclic mastalgia of two years duration. There was no other significant past history. Patient had undergone abdominal hysterectomy 4 years back for dysfunction uterine bleeding and was on hormone replacement therapy with estrogen alone for the same duration.

On examination there was 4 × 3 cm lump in upper outer quadrant of the right breast with no fixity to skin or underlying tissue. There were no significant axillary or supraclavicular nodes. Abdominal examination failed to show any organomegaly. Routine hematological, biochemical tests, chest roentgenogram, abdominal ultrasonogram and bone scans were normal. Fine needle aspiration cytology revealed Infiltrating duct carcinoma.

**Figure 1:**
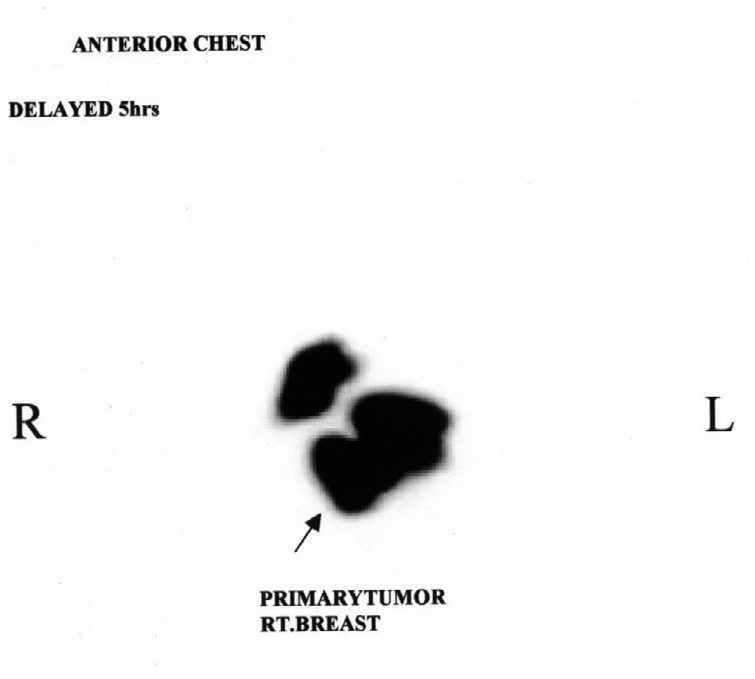
Lymphoscientigraphic scan showing radioneucleotide uptake in primary tumor, no sentinel node is identified.

Patient was planned for sentinel lymph node biopsy follow by mastectomy. 8 ml of Tc^99 ^sulfur colloid was injected around the tumor and immunoscintigraphy images (anterior and lateral view) were taken. These images failed to show any lymph node uptake either in axilla or else where (figure 1) at the time of surgery 4 ml of isosulfan blue was injected peritumorilly and 20 minutes later axilla was entered. Sentinel node detection was also carried out using a hand held gamma probe (Navigator^®^, Auto sutures). Sentinel lymph node was identified by combined technique in level I axilla lying just posterior to the primary tumor. On gross examination most of the node appeared to be replaced by tumor and only a part of it appeared normal this part was stained blue while rest of the node was white. Histopathology of primary tumors was infiltrating duct carcinoma, with involvement of skin. Sentinel lymph node showed metastatic deposit. Other lymph nodes in axillary dissection specimen were also positive.

### Case 2

A 42-year-old female presented with lump in left breast of 1-month duration. She was a known case of carcinoma breast and had undergone right modified radical mastectomy 7 years back followed by chest wall radiotherapy and 6 cycles of CMF chemotherapy.

On examination there was a 3 × 2 cm lump located in the retro areolar area of left breast. Scar of right mastectomy was seen. There were no palpable axillary nodes or supra clavicular nodes. Systemic examination was normal. Routine hematological and biochemical investigations chest x-ray, abdominal sonography and bone scans were normal. The treatment options were discuss with the patient and she wanted to conserve this breast and hence a wide excision of mass encompassing nipple areola complex, sentinel node biopsy followed by axillary clearance was planned.

**Figure 2:**
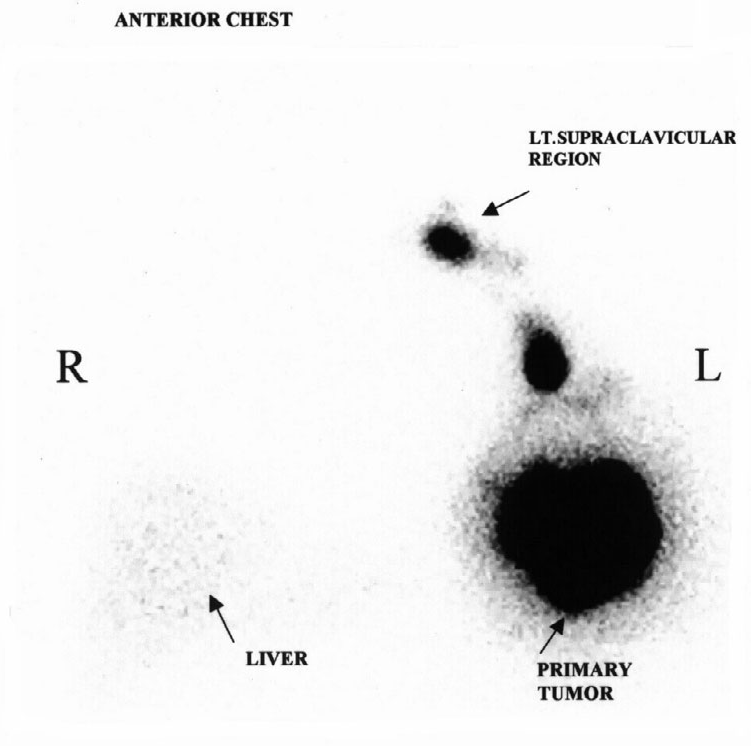
Lymphoscintigraphic scan showing uptake in primary tumor with sentinel node in axilla and in left supra clavicular area.

On the morning of the surgery 4 ml of Tc^99 ^sulfur colloid was injected peritumorily and scintigraphic images were taken 4 hours later. The scintigraphic image showed one sentinel node in axilla and other in supra clavicular fossa (figure 2). At surgery 4 ml of isosulfan blue was injected peritumorilly and sentinel node identification was carried out by combined method. On exploration of axilla the blue and hot sentinel node was identified and removed. However, hand held probe failed to pick a hot spot in supra clavicular fossa axillary dissection was completed.

Histopathology of the resected specimen showed infiltrative duct carcinoma margins of resection were negative. The sentinel node showed deposits from infiltrating duct carcinoma. Post operative period was uneventful and it is planned to start her on radiotherapy to residual breast with 4 cycles of anthracyclin based chemotherapy.

## Discussion

The axillary dissection for axillary nodes from breast cancer is still the standard of care its routine use in node negative breast cancer has been questioned due to morbidity associated with the axillary dissection. SLNB has improved the morbidity in patients with node negative breast cancer, while providing the much needed prognostic information. Although these techniques have been successful, they are still evolving, and SLN biopsy is not yet considered the standard of care in breast cancer.

Preoperative visualization is one of the three methods commonly employed in detection of sentinel node. When radioactive colloid is used, a preoperative lymphoscintigram often is obtained to ease SLN identification further. This has been reported to have a false negative rate of 3–30% in various series [11-13]. Several factors like age, size of the breast, presence of metastasis, neoadjuvant chemotherapy, has been proposed to influence the non-visualization [11-19]. Other authors have found no significant predictor of non visualization [20]. The non-visualization in our first case was due to superimposition of locally advance tumor in the outer quadrant and hence separation was not achieved on scintigraphy, while the erroneous supra clavicular node in the other was probably due to the spillage of radioactive material at the time of injection, which was subsequently washed off during the part preparation and hence no signal was obtained at intraoperative gamma probe assisted SLNB.

## Conclusion

These two cases demonstrate the importance of using the triple technique to maximize the identification of SLN and improve the sensitivity and specificity of SLNB and fallacies of preoperative lymphoscintigraphy. This also raises a question that should preoperative scintigraphy should be carried out in all the cases?

## Competing interests

The author(s) declare that they have no competing interests.

## Authors' contributions

**MP**: Conceived the idea, carried out the literature search, prepared the draft manuscript and edited it for publication.

**SVSD**: helped in preparation of the manuscript and edited the final version

**RM**: Carried out the preoperative lymphoscintigraphy and helped in preparation of the manuscript.

MP and SVSD carried out the intraoperative scintigraphy and managed the patients.

All authors read and approved the final manuscript.
